# Screening and Validation of Stable Reference Genes for qRT-PCR Analysis in *Epicauta gorhami* (Coleoptera: Meloidae)

**DOI:** 10.3390/insects15120942

**Published:** 2024-11-29

**Authors:** Guofeng Yang, Xuetao Yu, Yan Zhang, Jinhua Luo, Xiaofei Li, Li Zhu, Huanhuan Zhang, Lin Jin, Gang Wu, Xiaohong Yan, Chenhui Shen

**Affiliations:** 1 Key Laboratory of Agricultural Genetically Modified Organisms Traceability, Oil Crops Research Institute of Chinese Academy of Agricultural Science/Supervision and Test Center (Wuhan) for Plant Ecological Environment Safety, Ministry of Agriculture and Rural Affairs, Wuhan 430062, China; yangguofeng8866@163.com (G.Y.); xuetao.yu@outlook.com (X.Y.); zy18736825518@163.com (Y.Z.); lixiaofei@caas.cn (X.L.); zhuli49751@163.com (L.Z.); wugang@caas.cn (G.W.); 2 Enshi Tujia and Miao Autonomous Prefecture Academy of Agricultural Sciences, Enshi 445000, China; chw63@126.com; 3 Institute of Vegetable, Tibet Academy of Agricultural and Animal Husbandry Sciences, Lhasa 850032, China; 15365182126@163.com; 4 Department of Entomology, College of Plant Protection, Nanjing Agricultural University, Nanjing 210095, China; jinlin@njau.edu.cn

**Keywords:** *Epicauta gorhami*, *Glycine max*, reference genes, qRT-PCR, RefFinder

## Abstract

*Epicauta gorhami* is a hypermetamorphic insect that mainly forage soybeans during the adult stage. However, the lack of appropriate references hinders our studying of the gene function in *E. gorhami*. In this study, referring to five computational tools (Ct value, geNorm, NormFinder, BestKeeper and RefFinder), the stability of 10 housekeeping genes (*GAPDH*, *ACT*, *RPL4*, *RPL27*, *α-TUB*, *RPS18*, *EF1α*, *RPS28*, *RPL13* and *SOD*) was assessed by qRT-PCR under three different conditions (adult ages, tissues/organs and temperatures). The findings suggested that *SOD* and *RPS18* were the most ideal references for examine gene transcripts among diverse adult ages and at various temperatures; a pair of *RPS18* and *RPS28* was the most reliable genes to assess gene expressions in diverse adult tissues. Finally, the relative expression levels of *EgUAP* were computed after normalization *RPS18* and *RPS28* with across diverse adult tissues. As expected, *EgUAP* expression was abundant in the foregut, trachea and antenna and scarce in the midgut, hindgut and epidermis. These findings will lay a solid foundation for analyzing the gene expression of *E. gorhami*.

## Simple Summary

The reverse transcription-quantitative polymerase chain reaction (qRT-PCR) is often employed to examine the gene expressions under diverse treatments. Screening optimal reference genes are necessary for obtaining reliable expression results of qRT-PCR. Here, the stability of 10 selected reference genes in *Epicauta gorhami* was assessed under three conditions (adult ages, tissues/organs and temperatures). These findings displayed that the best suitable reference genes were as follows: *SOD* and *RPS18* for different adult ages and various temperatures and *RPS18* and *RPS28* for adult tissues. The relative expression patterns of uridine diphosphate (UDP)-N-acetylglucosamine-pyrophosphorylase (*EgUAP*) in diverse adult tissues was employed to verify the results. Our study will lay a vital basis for future functional gene expressions in *E. gorhami*.

## 1. Introduction

The bean blister beetle, *Epicauta gorhami* (Coleoptera: Meloidae) is a hypermetamorphic insect that can forage soybeans (*Glycine max*) during the adult stage [1,2,3,4]. It poses potential threats to soybean production, which is a crucial oilseed, protein and biodiesel crop [5,6]. The adults of this species mainly feed on leaves of *G. max* [1,2,3,4]. Previous studies on *E. gorhami* have traditionally placed a strong emphasis on diapause [1,3], seasonal adaptation [2], mitogenomic sequencing [7] and the phylogenetic relationship [4,7]. However, there is currently no research on gene functions in *E. gorhami*. In order to further explore molecular mechanisms of target genes, it is imperative to screen the suitable reference genes.

The reverse transcription-quantitative polymerase chain reaction (qRT-PCR) has advantages of high sensitivity, specificity and accuracy, which is widely employed to analyze the target gene transcripts [8,9]. For accurately measuring the mRNA levels of target genes by qRT-PCR, selecting the most optimal references to conduct normalization is essential [10,11]. If unstable reference genes are employed, the results of gene transcripts will be inaccurate [9,10]. Hence, selecting appropriate reference genes should be assessed under various experimental conditions [12,13,14].

As usual, housekeeping genes (HKGs), such as actin (*ACT*), ribosomal protein (*RPL* and *RPS*) and elongation factor 1α (*EF1α*), are widely employed as references in insect species, due to relative stable expression levels. However, the stability of internal genes may not always constant under diverse experimental treatments [14,15]. In the potato ladybird *Henosepilachna vigintioctomaculata*, *RPL6* and *RPL13* are the suitable references in different developmental stages, whereas *RPS18* and *EF1α* are more optimal among diverse tissues [14]. In the sugarcane stem borer *Chilo sacchariphagus*, *β-ACT* and *RPL7* show the best stable expression in different tissues, whereas *EF1α* and *SDHA* exhibit stability between sexes [15]. Hence, evaluating reference genes at different backgrounds is necessary.

Currently, important advances have been made in the study of reference genes selection in numerous insect species, such as *Mylabris sibirica* (*RPL6* and *RPL13*) [12], *Phthorimaea operculella* (*EF1α* and *RPL13*) [13], *H. vigintioctomaculata* (*RPS18* and *RPL13*) [14] and *C. sacchariphagus* (*β-ACT* and *RPL7* across various tissues and at distinct temperatures; *EF1α* and *SDHA* between sexes) [15]. To sum up, each experimental condition needs at least two reference genes to measure the target gene transcripts in insect species.

In this paper, we selected 10 potential reference genes from the full-length transcriptome of *E. gorhami* adults (unpublished data), which were *ACT*, *EF1α*, *GAPDH* (glyceraldehyde-3-phosphate dehydrogenase), *α-TUB* (α-tubulin), *RPL4*, *RPL13*, *RPL27*, *RPS18*, *RPS28* and *SOD* (superoxide dismutase). The stability of these genes was assessed at diverse adult ages, across different adult tissues and at various temperatures of *E. gorhami*. In addition, the expression pattern of *E. gorhami* uridine diphosphate (UDP)-N-acetylglucosamine (UDP-GlcNAc) pyrophosphorylase (*EgUAP*) across different adult tissues was used to evaluate the results. These findings will lay a vital basis for further research on gene functions in *E. gorhami*.

## 2. Materials and Methods

### 2.1. Insect Rearing and Treatment Methods

*E. gorhami* adults used in this study were obtained in August 2024 from *G. max* plants in Enshi Tujia and Miao Autonomous Prefecture, Hubei Province, China (coordinates: 30°19′43″ N, 109°38′48″ E). The adults were fed under suitable conditions (25 ± 2 °C, 16 h:8 h photoperiod and 50–60% relative humidity) in the insectary for one week.

Adult ages: Four newly emerged adults (two males and two females) were sampled for one biological replicate, every day for a continuous period of 3 days (1-day-old adults, 2-day-old adults and 3-day-old adults). Three biological replicates were conducted.

Adult tissues: These tissues (foregut, midgut, hindgut, antenna, trachea and epidermis) were collected from the 5-day-old adults. Ten adults (5 males and 5 females) were dissected for one biological replicate. Three biological replicates were conducted.

Temperature: The newly emerged adults were reared at three temperatures (4 °C, 25 °C and 37 °C) for 6 h. Four newly emerged adults (2 males and 2 females) were treated for one biological replicate. Three biological replicates were prepared.

### 2.2. Total RNA Isolation and cDNA Synthesis

Total RNA of each sample was extracted by the TRIzol reagent (YiFeiXue Tech, Nanjing, China), referring to the manufacturer’s instructions. The RNA’s purity and concentration were detected by a NanoDrop ND-1000 spectrophotometer. Subsequently, 1000 ng of total RNA was used to synthesize cDNA using the HiScript III RT SuperMix with gDNA wiper (Vazyme Biotech Co., Ltd., Nanjing, China). The reaction mixtures were subjected to incubation at 37 °C for 15 min, followed by 85 °C for 5 s. The resultant cDNA samples were diluted 3-fold for the pursuant PCR and qRT-PCR.

### 2.3. Selection of Candidate References and Primer Design

Based on the full-length transcriptome of *E. gorhami* adults, the BioEdit software (version 7.1.3.0) was employed to conduct a TBLASTN search by using the amino acid sequence of ten candidate reference genes as the queries from *M. sibirica* [12] and *P. operculella* [13]. This resulted in the identification of ten reference genes in *E. gorhami*. These genes included *EF1α*, *ACT*, *RPL4*, *α-TUB*, *RPL13*, *RPL27*, *RPS18*, *GAPDH*, *RPS28* and *SOD*, all of which are commonly utilized as reference genes in other insects [16].

The correctness of the reference gene sequences was substantiated by polymerase chain reaction (PCR) using primers in Table S1. Primer pairs were designed to verify the complete open reading frames of reference genes by Primer Premier 5.0 software (version 5.00), based on the RT-PCR primer design principles [15]. After the amplicons of RT-PCR were confirmed by Sanger sequencing, the sequences of amplicons were uploaded to GenBank, with the accession numbers of PQ497541-PQ497541 (Table S1).

### 2.4. Quantitative Real-Time PCR (qRT-PCR)

The primers for qRT-PCR were performed by Primer3web version 4.1.0 (https://primer3.ut.ee/, accessed on 12 September 2024) and are presented in Table 1. The products of qRT-PCR were verified by Sanger sequencing. The qRT-PCR experiment was executed by a CFX96 Real-Time System (Bio-Rad Laboratories, Hercules, CA, USA) and ChamQ Universal SYBR qPCR Master Mix (Vazyme Biotech Co., Ltd.). Each reaction consisted of a final volume of 20 μL, containing 10 μL of 2 ChamQ Universal SYBR qPCR Master Mix, 0.4 μL of reverse primer (10 μM), 1 μL of cDNA template and 8.2 μL of RNase-free water. The thermocycling procedure consisted of an initial step of 95 °C for 30 s, followed by 40 cycles of 95 °C for 5 s and annealing at 60 °C for 34 s. After the reaction, a melting curve analysis was conducted for one cycle of 95 °C for 15 s, 60 °C for 60 s and 95 °C for 1 s, which was used to confirm the specificity of the amplified product. The amplification efficiency (E) was computed using a series of 3-fold dilutions of a cDNA template, referring to the equation E = (10^[−1/slope]^ − 1) × 100% [17]. All experiments were conducted in triplicate.

### 2.5. Assessment of Reference Gene Stability

The raw Ct values were required for the BioRadCFXManager software (version 3.1.1517.0823). The stability of 10 reference genes under different backgrounds were assessed using a range of algorithms, including BestKeeper [18], Normfinder [19], geNorm [10] and the ΔCt method [20]. In addition, the suitable number of reference genes under different conditions was confirmed by pairwise variation (Vn/n + 1), required for the GeNorm method. In general, a Vn/n + 1 value below the threshold of 0.15 suggests that the starting n reference genes are suitable to normalize gene expression. Finally, the RefFinder [21,22] was applied to analyze the comprehensive ranking of each experimental condition.

### 2.6. Validation of Selected Reference Genes in Diverse Adult Tissues

The transcription level of uridine diphosphate (UDP)-N-acetylglucosamine-pyrophosphorylase (*UAP*, GenBank: PQ497551) in *E. gorhami* was utilized to assess the stability of selected reference genes across various adult tissues. The primer sequence of *UAP* was as follows: Forward: CGCTACAACGTAACGCCATC, Reverse: CCCCACAATCGCTACGTTTC. The relative transcription level of *EgUAP* in adult tissues were computed by the 2^−∆∆Ct^ method [23], based on *RPS18* and *RPS28*. The SPSS Statistics 29 software (version 29.0.2.0) package was then used to evaluate the variance in expression levels of *EgUAP* among different adult tissues through one-way ANOVA analysis followed by Tukey’s test for multiple comparisons.

## 3. Results

### 3.1. Selection of Candidate Reference Genes

According to the full-length transcriptome of *E. gorhami*, we applied the BioEdit software (version 7.1.3.0) to perform a TBLASTN search by using the amino acid sequence of ten candidate reference genes as the queries from *M. sibirica* [12] and *P. operculella* [13]. This enabled the identification of ten cDNAs, which we named *ACT*, *GAPDH*, *RPL4*, *EF1α*, *RPL13*, *RPL27*, *RPS18*, *RPS28*, *α-TUB* and *SOD* in *E. gorhami*. We used the Primer Premier 5.0 software to design the primers, with the lengths of 18–22 bp. After the amplification round and then Sanger sequencing, the sequencing results of amplicons were uploaded to GenBank under these accession numbers: PQ497541-PQ497541 (Table S1).

The Primer3web version 4.1.0 (https://primer3.ut.ee/, accessed on 12 September 2024) was used to design the qRT-PCR primers, with the amplification product length ranging from 80 bp to 250 bp. The products of qRT-PCR were verified by sequencing. The melting curve analysis confirmed the specificities of each primer pair for qRT-PCR. As expected, the efficiency (*E*) of 10 primer pairs were between 95.50% (*EF1α*) and 102.73% (*α-TUB*), with the correlation coefficients (R^2^) varying from 0.993 to 0.999 (Table 1). These results demonstrated that efficiency of primers reached the standards of traditional qRT-PCR [24].

### 3.2. Expression Levels of Reference Genes

**Figure 1:**
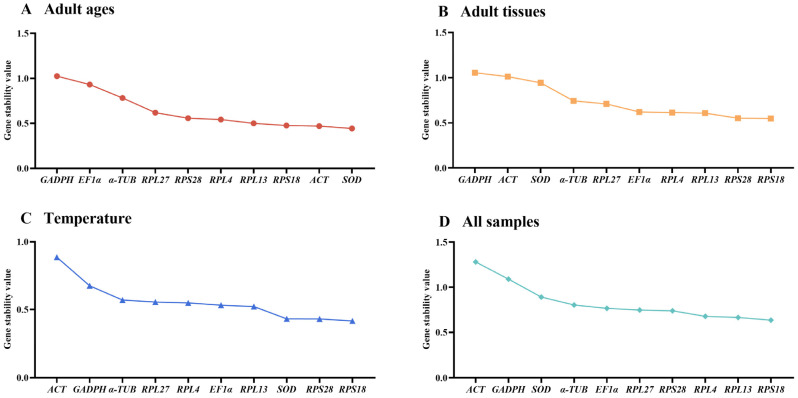
Stability rankings of the ten candidate reference genes in *Epicauta gorhami* computed by the ΔCt method in different samples. The stability values are showed from the least (**left**) to the most stable gene (**right**). Abbreviation: ACT, actin; SOD, superoxide dismutase; α-TUB, α-tubulin; GAPDH, glyceraldehyde-3-phosphate dehydrogenase; EF1α, elongation factor 1α; RPL4, RPL13, RPL27, RPS18 and RPS28, ribosomal protein. The abbreviations are exactly the same as Figures 2–5.

The threshold-cycle (Ct) of ten reference genes under various testing conditions were presented (Figure 1, Table S2). Among diverse adult ages, *SOD* exhibited smaller gene expression variations, whereas *GAPDH* and *EF1α* demonstrated greater fluctuations (Figure 1A). Across different adult tissues and under different temperature treatments, the expression fluctuations were lower in *RPS18* and *RPS28* and higher in *ACT* and *GAPDH* (Figure 1B,C). A combination of above results indicated that the expression difference was small in all reference genes except for *ACT* and *GAPDH* (Figure 1D).

### 3.3. Stability of the Ten HKGs Among Adult Ages

**Figure 2:**
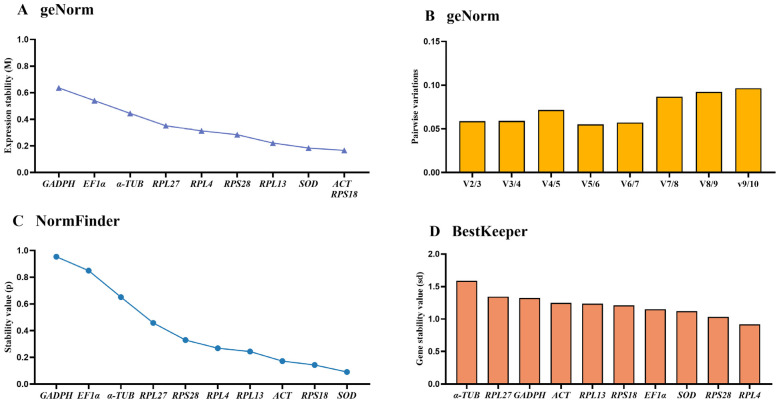
Stability rankings of the ten candidate reference genes in *Epicauta gorhami* computed by geNorm, NormFinder and BestKeeper at various adult ages. Diverse ages of *Epicauta gorhami* adults were sampled (collected on the first to third days of the newly emerged adults). The stability values are showed from the least stable (**left**) to the most stable gene (**right**). (**A**,**B**) geNorm, (**C**) NormFinder and (**D**) BestKeeper.

Based on average expression stability (M-values) and pairwise variation (V-values), the geNorm algorithm evaluates gene stability. These results exhibited that *ACT*, *RPS18* and *SOD* were the most suitable, with M-values below 0.20 (Figure 2A, Table 2). Moreover, V-values suggested that V2/3 to V9/10 values were below 0.15, showing that two diverse references were equal for assessing the gene transcript among adult ages (Figure 2B).

The NormFinder algorithm indicated that the stable ranking of ten reference genes from the most to the least stable were as follows: *SOD*, *RPS18*, *ACT*, *RPL13*, *RPL4*, *RPS28*, *RPL27*, *α-TUB*, *EF1α* and *GAPDH*, with the *p*-value of 0.091, 0.143, 0.172, 0.243, 0.268, 0.329, 0.458, 0.651, 0.849 and 0.954, respectively (Figure 2C, Table 2). The BestKeeper analysis showed that RPL4, RPS28 and SOD were the top three stable genes (Figure 2D, Table 2).

Referring to the results of RefFinder, the ranking of 10 reference genes at adult ages were as follows: *SOD* > *RPS18* > *ACT* > *RPL4* > *RPS28* > *RPL13* > *EF1α* > *RPL27* > *α-TUB* > *GAPDH* (Figure 5A). Therefore, the combination of SOD and RPS18 are the most appropriate for gene transcript analysis by qRT-PCR among various adult ages (Table 3).

### 3.4. Stability of the Ten HKGs Across Various Adult Tissues

**Figure 3:**
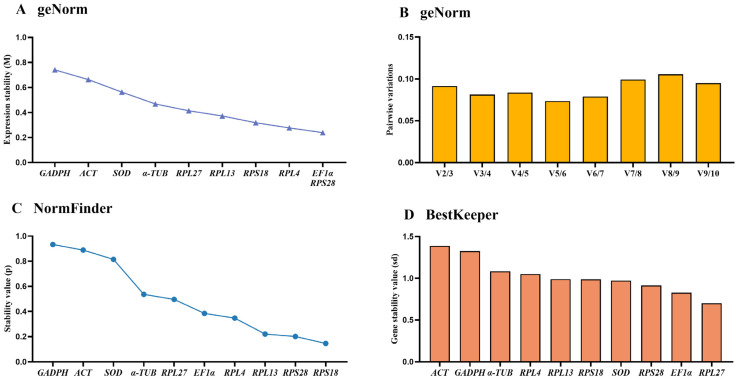
Stability rankings of the ten candidate reference genes in *Epicauta gorhami* calculated by geNorm, NormFinder and BestKeeper among diverse adult tissues. Foregut, midgut, hindgut, epidermis, trachea and antenna were dissected from the 5-day-old adults. The stability values are showed from the least stable (**left**) to the most stable gene (**right**). (**A**,**B**) geNorm, (**C**) NormFinder and (**D**) BestKeeper.

The geNorm results displayed that *RPS28*, *EF1α*, *RPL4* and *RPS18* were the top four stable genes (Figure 3A, Table 2). V-values data demonstrated that all values of from V2/3 to V9/10 were below 0.15, showing that two reference genes in various adult tissues were suitable (Figure 3B).

According to the NormFinder data, the rankings of reference genes were as follows: *RPS18* > *RPS28* > *RPL13* > *RPL4* > *EF1α* > *RPL27* > *α-TUB* > *SOD* > *ACT* > *GAPDH* (Figure 3C, Table 2). Moreover, the *p*-values of all reference genes were less than 1.0 (Figure 3C, Table 2). The BestKeeper results indicated that *RPS18* and *RPS28* were the most stable, with the SD values of 0.700 and 0.914, respectively (Figure 3D, Table 2). Furthermore, the SD values of *ACT*, *GADPH*, *α-TUB* and *RPL4* were above 1.0 (Figure 3D, Table 2).

According to RefFinder results, the comprehensive ranking order were as follows: *RPS28* > *RPS18* > *EF1α* > *RPL27* > *RPL13* > *RPL4* > *SOD* > *α-TUB* > *ACT* > *GAPDH* (Figure 5B). Therefore, the pair of *RPS18* and *RPS28* is the most appropriate for qRT-PCR data normalization among diverse adult tissues (Table 3).

### 3.5. Stability of the Ten HKGs Under Diverse Temperature Conditions

**Figure 4:**
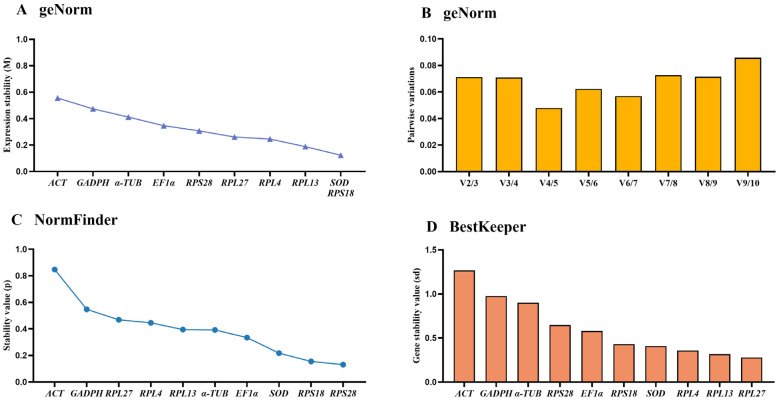
Stability rankings of the ten candidate reference genes in *Epicauta gorhami* assessed by geNorm, NormFinder and BestKeeper at different temperatures. The newly emerged adults reared for 6 h under three temperatures (4 °C, 25 °C and 37 °C) were collected. The stability values are showed from the least stable (**left**) to the most stable gene (**right**). (**A**,**B**) geNorm, (**C**) NormFinder and (**D**) BestKeeper.

The geNorm results indicated that *RPS18*, *SOD* and *RPL13* were the steadiest references in diverse temperatures, with M-values of 0.123, 0.123 and 0.188, respectively (Figure 4A, Table 2). Moreover, V-values data displayed that all values were below 0.15, showing that two reference genes under various temperature treatments were suitable (Figure 4B).

The NormFinder results manifested that the steady rankings were *RPS28*, *RPS18*, *SOD*, *EF1α*, *α-TUB*, *RPL13*, *RPL4*, *RPL27*, *GAPDH* and *ACT* (Figure 4C, Table 2). BestKeeper data uncovered that the SD values of these genes were less than 1.0, except for *ACT* (Figure 4D, Table 2).

**Figure 5:**
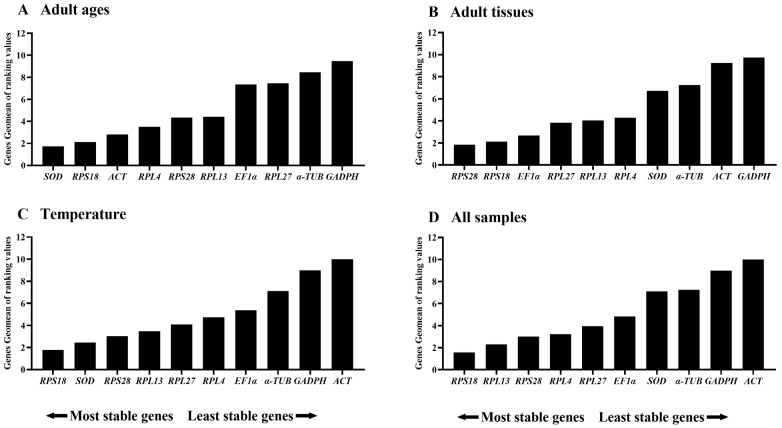
Stability rankings of the ten candidate reference genes in *Epicauta gorhami* computed by the Geomean method of RefFinder in different samples. The stability values are showed from the most stable (**left**) to the least stable gene (**right**). (**A**) adult ages, (**B**) adult tissues, (**C**) temperature and (**D**) all samples.

Refer to the results of RefFinder, the stability orders were as follows: *RPS18* > *SOD* > *RPS28* > *RPL13* > *RPL27* > *RPL4* > *EF1α* > *α-TUB* > *GAPDH* > *ACT* (Figure 5C). To sum up, RPS18 and SOD are the best reference gene pair to evaluate the gene transcript under diverse temperature conditions (Table 3). When combining the three diverse treatments together, the RefFinder results showed that the stability ranking in sequence were *RPS18*, *RPL13*, *RPS28*, *RPL4*, *RPL27*, *EF1α*, *SOD*, *α-TUB*, *GAPDH* and *ACT* (Figure 5D).

### 3.6. Validation of the Selected Reference Genes

**Figure 6:**
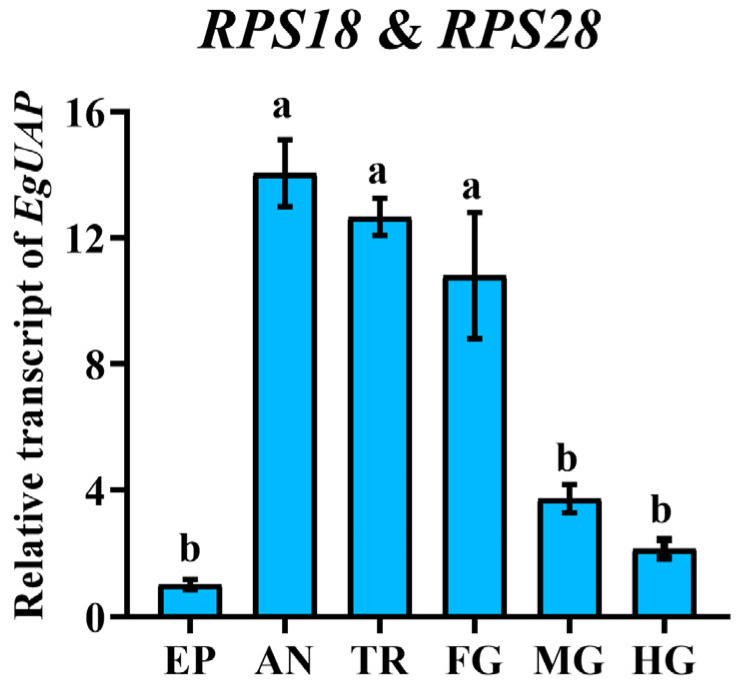
Relative expression level of *EgUAP* among diverse adult tissues of *Epicauta gorhami*. The relative gene expression level of *EgUAP* in the foregut (FG), midgut (MG), hindgut (HG), epidermis (EP), trachea (TR) and antenna (AN) was normalized to the most suitable reference genes (*RPS18* and *RPS28*) by the 2^−∆∆Ct^ method. The relative transcripts are the ratios of copy numbers in different adult tissues relative to the epidermis, which is set at 1. The values are means + SE. Different letters indicate significant differences in gene expression among different tissues (*p* < 0.05).

To estimate the performance of the recommended reference genes, the relative mRNA level of *EgUAP* in various adult tissues was measured using the most stable references (*RPS18* and *RPS28*) by the 2^−∆∆Ct^ method [23]. Our results indicated that the transcription level of *EgUAP* was high in the foregut, trachea and antenna and low in the midgut, hindgut and epidermis of *E. gorhami* (Figure 6).

## 4. Discussion

In this study, the expression stabilities of ten selected reference genes in *E. gorhami* were estimated to perform qRT-PCR analysis normalization, laying a vital basis for future study of gene functions. Notably, we evaluated the stability of these genes under three different conditions (adult ages, adult tissues/organs and temperatures) for the first time in *E. gorhami*. These findings suggested that *SOD* and *RPS18* were the most ideal reference combination to measure gene transcription levels among different adult ages (Figure 1, Figure 2 and Figure 5 and Table 2 and Table 3) and at various temperatures (Figure 1, Figure 4 and Figure 5 and Table 2 and Table 3); *RPS18* and *RPS28* was the most reliable genes to assess gene expressions under diverse adult tissues (Figure 1, Figure 3 and Figure 5 and Table 2 and Table 3).

Here, we used four programs (geNorm, Normfinder, Bestkeeper and ∆CT) to examine the suitability of reference genes. The findings suggest that different programs’ results under different experimental conditions present diverse most stable reference genes (Figure 1, Figure 2, Figure 3 and Figure 4). For example, in different adult tissues, the geNorm method showed *EF1α* and *RPS28* were the most stable reference genes, and the Normfinder and ∆CT methods identified *RPS18* as the top-ranking reference gene, while the Bestkeeper ranked *RPL27* as the most stable reference gene (Figure 1 and Figure 3, Table 2). These differences may be due to diverse statistical algorithms of each method, which can be found in the publications [10,18,19,20]. Under such a condition, RefFinder, a web-based tool is widely used to compute the geometric mean of the rankings obtained by the different algorithms, which solves the limitations of operating one single program [21,22].

Ribosomal proteins play a vital function on ribosome assembly, which bind to four ribosomal RNAs (rRNAs) to constitute the ribosomes [25]. Similar to our findings, ribosomal proteins are selected as the most optimal reference genes in insect species, such as Coleopterans *M. sibirica* (*RPL6* and *RPL13*) [12], *Leptinotarsa decemlineata* (*RP18* and *RP4*) [26], *Phaedon brassicae* (*RPL32* and *RPL19*) [27], *Henosepilachna vigintioctopunctata* (*RPL13* and *RPS18*) [16], *Tribolium castaneum* (*RPS6*, *RPL13a*, *RPS3* and *RPL18*) [28], *Ips sexdentatus* (*RPS3*) [29] and *H. vigintioctomaculata* (*RPS18* and *RPL13*) [14]; Hemipterans *Psammotettix striatus* (*RPLP2*) [30], *Rhopalosiphum padi* (*RPL13*, *RPS6* and *RPS18*) [31], *Aphis glycines* (*RPS9*) [32], *Ferrisia gilli* (*RPS8*, *RPL40* and *RPL7*) [33], *Diaphorina citri* (*RPL7*) [34] and *Dichelops melacanthus* (*RPL9* and *RPS23*) [35]; Hymenopterans *Anastatus japonicus* (*RPL13* and *RPS6*) [36]; Lepidopterans *Mythimna loreyi* (*RPL10*, *RPL27* and *RPS3*) [37], *Plutella xylostella* (*RPS13* and *RPS23*) [38], *Spodoptera litura* (*RPS13* and *RPLP0*) [39], *P. operculella* (*RPL13*) [13], *Bombyx mori* (*RPS7*) [40] and *Helicoverpa armigera* (*RPS15* and *RPL27*) [41]; Dipterans *Aphidoletes aphidimyza* (*RPL8* and *RPS3*) [42], *Exorista sorbillans* (*RP49*) [43] and *Chlorops oryzae* (*RPS15*) [44]; Orthopteran *Locusta migratoria* (*RPL32*) [45] and Thysanopterans *Megalurothrips usitatus* (*RPL30*) [46] and *Frankliniella occidentalis* (*RPL32*) [47].

At different adult ages and temperatures, *SOD* was verified to be the most reliable gene (Figure 1, Figure 2, Figure 4 and Figure 5 and Table 2 and Table 3). Superoxide dismutase (SOD) is a crucial antioxidant enzyme, catalyzing the conversion of reactive oxygen species into oxygen and hydrogen peroxide [48]. Consistent with our data, SOD is recommended as the most appropriate reference gene in *Spodoptera frugiperda* [49], *Thrips tabaci* [50], *Riptortus pedestris* [51] and *Spodoptera exigua* [52].

In addition, the BestKeeper analysis data indicated that the SD values of *α-TUB*, *EF1α*, *GAPDH* and *ACT* were greater than 1.0 (Table 2), showing that these genes were unaccommodated as reference genes to perform qRT-PCR normalization. Similar results have been verified in other insects, such as *P. operculella* [13], *Ophraella communa* [53], *A. aphidimyza* [42], *Hippodamia convergens* [54], *H. vigintioctomaculata* [14], *Colaphellus bowringi* [55], *H. vigintioctopunctata* [16] and *M. sibirica* [12].

To further validate the accuracy of *RPS18* and *RPS28* in qRT-PCR normalization in *E. gorhami*, we examined the relative transcript level of *EgUAP* in diverse adult tissues. Our results exhibited that *EgUAP* expression was high in the foregut, trachea and antenna and low in the midgut, hindgut and epidermis (Figure 6). The expression pattern is consistent with the fact that UAP catalyzes the formation of UDP-GlcNAc, which is the precursor of the production of chitin in ectodermally derived epidermal cells, foregut, hindgut and trachea [56,57,58].

Overall, it is essential to screen and verify the most suitable references to guarantee the accuracy of gene expression. This study would offer a solid basis for further molecular functions of target genes in *E. gorhami*.

## 5. Conclusions

Ten potential reference genes in *E. gorhami* were assessed for accurate qRT-PCR analysis of *E. gorhami* under three different treatments. These results demonstrated that the steadiest reference genes were as follows: *SOD* and *RPS18* for different adult ages and various temperatures and *RPS18* and *RPS28* for adult tissues. This study is the first one to establish the qRT-PCR normalization analyses in *E. gorhami*, facilitating further research on gene functions of *E. gorhami*.

## Figures and Tables

**Table 1:** Primers of 10 candidate house-keeping genes used in qRT-PCR.

Gene	Primer Sequences (5′to 3′)	Length (bp)	Slope	R^2^	Efficiency (%)
*EF1α*	F-ATCATTGACGCACCTGGACA	99	−3.435	0.999	95.50
	R-ACCAGTACCAGCAGCAACAA				
*GAPDH*	F-ACAGTACATGCCACCACAGC	180	−3.351	0.999	98.82
	R-TGGTACACGGAAGGCCATAC				
*RP* *L4*	F-TCGATGAACCACCGTCAACC	80	−3.308	0.993	100.60
	R-CGACCGGTACCCCATGATTC				
*RPL13*	F-AAGCCGCCGGTATTAACAGC	172	−3.318	0.998	100.16
	R-TCACCAGGACGCAACTTCTT				
*RPL27*	F-TCGTATTGGTCTTGGCAGGC	112	−3.373	0.999	98.03
	R-TCAATGCCGGCAACAATAGC				
*SOD*	F-AGTTGTCCATGCTGATCCGG	95	−3.32	0.998	100.08
	R-TAACACCACAGGCCAAACGT				
*ACT*	F-TACGTGTGGCACCTGAAGAA	169	−3.284	0.997	101.60
	R-CCAGTTGTACGACCAGAAGCA				
*α-TUB*	F-ATGGGCACGTCTTGATCACA	174	−3.258	0.999	102.73
	R-TCATTTTCACCTTCGCCCGA				
*RPS18*	F-AGGTGTTGGTCGTCGTTACTC	210	−3.323	0.999	99.96
	R-TCTAAGGTAGCCGATGTGAGC				
*RPS28*	F-GGTGAGCAAAACCGTCAGATC	90	−3.347	0.997	98.97
	R-TGCTTCACGTTCAGACTCCA				

Note: ACT, actin; SOD, superoxide dismutase; α-TUB, α-tubulin; GAPDH, glyceraldehyde-3-phosphate dehydrogenase; EF1α, elongation factor 1α; RPL4, RPL13, RPL27, RPS18 and RPS28, ribosomal protein.

**Table 2:** Stability rankings of the ten reference genes under diverse experimental backgrounds.

Conditions	CRGs *	geNorm		Normfinder		BestKeeper		ΔCt	
		M-Value	Rank	*p*-Value	Rank	Stability	Rank	Stability	Rank
Adult ages	*EF1α*	0.540	8	0.849	9	1.149	4	0.931	9
	*GADPH*	0.636	9	0.954	10	1.325	8	1.024	10
	*RPL4*	0.313	5	0.268	5	0.920	1	0.543	5
	*RPL13*	0.221	3	0.243	4	1.236	6	0.500	4
	*RPL27*	0.351	6	0.458	7	1.344	9	0.619	7
	*SOD*	0.184	2	0.091	1	1.121	3	0.443	1
	*ACT*	0.166	1	0.172	3	1.249	7	0.469	3
	*α-TUB*	0.444	7	0.651	8	1.589	10	0.782	8
	*RPS18*	0.166	1	0.143	2	1.209	5	0.476	2
	*RPS28*	0.284	4	0.329	6	1.033	2	0.557	6
Adult tissues	*EF1α*	0.239	1	0.384	5	0.828	2	0.621	5
	*GADPH*	0.741	9	0.933	10	1.326	9	1.055	10
	*RPL4*	0.277	2	0.347	4	1.051	7	0.614	4
	*RPL13*	0.372	4	0.220	3	0.989	6	0.608	3
	*RPL27*	0.414	5	0.496	6	0.700	1	0.710	6
	*SOD*	0.563	7	0.815	8	0.971	4	0.944	8
	*ACT*	0.663	8	0.889	9	1.389	10	1.012	9
	*α-TUB*	0.468	6	0.536	7	1.085	8	0.744	7
	*RPS18*	0.318	3	0.146	1	0.987	5	0.549	1
	*RPS28*	0.239	1	0.201	2	0.914	3	0.552	2
Temperature treatment	*EF1α*	0.346	6	0.334	4	0.58	6	0.532	5
	*GADPH*	0.474	8	0.547	9	0.976	9	0.675	9
	*RPL4*	0.246	3	0.446	7	0.357	3	0.549	6
	*RPL13*	0.188	2	0.395	6	0.317	2	0.522	4
	*RPL27*	0.261	4	0.468	8	0.278	1	0.555	7
	*SOD*	0.123	1	0.217	3	0.407	4	0.431	3
	*ACT*	0.556	9	0.847	10	1.265	10	0.886	10
	*α-TUB*	0.412	7	0.392	5	0.899	8	0.570	8
	*RPS18*	0.123	1	0.155	2	0.429	5	0.415	1
	*RPS28*	0.307	5	0.131	1	0.646	7	0.430	2

∗ Candidate reference gene.

**Table 3:** The most optimal reference genes in *E. gorhami* for various experimental backgrounds.

Experimental Conditions	The Recommended Reference Genes	
Adult ages	*SOD*	*RPS18*
Adult tissues	*RPS28*	*RPS18*
Temperature	*SOD*	*RPS18*

## Data Availability

The data presented in this study are included in the article and Supplementary Materials; further inquiries can be directed to the corresponding authors.

## Supplementary Materials

The following supporting information can be downloaded at: https://www.mdpi.com/article/10.3390/insects15120942/s1, Table S1: A list of primers used for RT-PCR of the genes and Table S2: The overall threshold cycle (Ct) values under different experimental conditions.
