# Supplementary material for: Screening and Validation of Stable Reference Genes for qRT-PCR Analysis in Epicauta gorhami (Coleoptera: Meloidae)
Source: Insects. 2024 Nov 29;15(12):942. doi: 10.3390/insects15120942 (PMC11678893; doi:10.3390/insects15120942)
Supplement: Supplementary file 1 [file insects-15-00942-s001.zip › insects-3307815-supplementary.pdf]

insects

MDPI

Supplementary Materials

Screening and Validation of Stable Reference Genes for qRT-PCR Analysis in *Epicauta gorhami* (Coleoptera: Meloidae)

Guofeng Yang [1,†], Xuetao Yu [1,†], Yan Zhang [1], Jinhua Luo [2], Xiaofei Li [1], Li Zhu [1], Huanhuan Zhang [3], Lin Jin [4], Gang Wu [1], Xiaohong Yan [1,*] and Chenhui Shen [1,*]

Table S1. A list of primers used for RT-PCR of the genes.

Gene name	Primer sequences (5'to 3′)	Amplicon size (bp)	Accession number
EF1α	Forward ATGGGTAAAGAAAAGACTCACA<br>Reverse CAGAACGACGGTCAACTTTT	1150	PQ497541
GADPH	Forward ATGTCTAAAATTGGTATCAACG<br>Reverse TTAATCTTTAGTTTGAATAAATTT	999	PQ497542
RPL4	Forward ATGTCGCTCGCGGCCGCT<br>Reverse ACTCGCTGCTGGTTTGCCTG	1272	PQ497543
RPL13	Forward ATGGCGCCGAAGGGGAATAATA<br>ReverseAGGTACCCAAGCTTTCGTTTTAATT	510	PQ497544
RPL27	Forward ATGGGTAAAATTATGAAATCTGG<br>ReverseTTAGAATCTAAGTTTTTGGAAAAAC	408	PQ497545
SOD	Forward ATGGCAACAAAAGCCGTATG<br>Reverse TTACAATTTGGTGATGCCG	465	PQ497546
ACT	Forward ATGTGTGACGAAGAAGTTGC<br>Reverse TTAGAAGCATTTCCTGTGG	1131	PQ497547
α-TUB	Forward ATGCGTGAATGTATCTCAGTCC<br>Reverse CTAATATTCTTCACCACCTTCA	1353	PQ497548
RPS18	Forward ATGTCTCTTGTAATTCCAGAAA<br>Reverse TTATTTCTTTTTGGATACACCA	459	PQ497549
RPS28	Forward ATGGATAAACCAGTCGTTTTG<br>Reverse TTATTTCTTTTTGGATACACCA	198	PQ497550

Note: EF1α, elongation factor 1α; GAPDH, glyceraldehyde-3-phosphate dehydrogenase; ACT, actin; SOD, Superoxide dismutase; α-TUB, α-tubulin; RPL4, RPL13 ,RPL27, RPS18 and RPS28, ribosomal protein.

Table S2. The overall threshold cycle (Ct) values under different experimental conditions.

Conditions / CRGs*	EF1α	GADPH	RPL4	RPL13	RPL27	SOD	ACT	α-TUB	RPS18	RPS28
**Adult ages**	21.06906	23.82622	23.41502	22.02107	22.24849	22.28670	23.64807	23.20798	22.23408	22.41215
	20.34058	23.61908	23.16868	21.93830	22.15568	22.05361	23.43063	22.95318	21.99412	22.31987
	20.88879	23.72916	23.82894	22.00665	22.10120	22.19839	23.51594	23.13086	22.05552	22.39687
	18.41774	20.88302	20.72629	18.39296	18.61830	18.85515	20.00251	18.90926	18.81579	19.56705
	18.11664	21.09159	20.82229	18.43586	18.59358	18.89785	20.08708	19.06181	18.36087	19.59345
	18.08785	20.96711	20.74089	18.61252	18.42515	18.89419	19.58687	19.30614	18.41255	19.58126
	21.41408	20.70913	22.13477	20.36288	21.17629	20.36227	21.50035	19.89211	20.14044	20.82320
	20.14230	20.48051	22.05417	20.17611	21.00933	20.49056	21.55613	19.91681	20.23069	20.84056
	19.14134	20.33735	22.18734	20.70899	20.72248	20.44645	21.59669	20.04235	20.27520	21.55615
Larvae tissues	18.96507	22.67228	21.61560	19.71238	19.89119	21.09128	21.32786	20.60650	19.79145	21.34726
	18.85646	23.06668	21.63664	20.91789	19.65340	20.99161	20.99164	20.49705	20.19377	21.10109

	19.17162	22.90798	22.1166120.3011319.4578821.0233021.3467021.0832920.15980	21.24514
	19.17289	19.25638	22.0763918.9485619.2013519.5709219.1932820.0655119.10568	21.04508
	19.24288	18.69916	21.8476719.2239319.4424419.9788319.5765820.3745619.21381	20.95391
	18.83837	19.76747	21.9174219.1014319.4502419.9365019.7651420.6681519.05506	21.06077
	18.05264	19.54123	20.3186618.1896019.3137718.3130819.1230018.6814217.81097	19.69861
	17.82929	20.03115	20.4062318.4704519.0081518.3754619.1748118.6327117.93718	19.86234
	17.67011	19.66895	20.3158218.7004018.8848018.3247319.2129318.7998417.91833	19.70371
	19.03250	21.01518	21.3980319.6694020.1471221.4413819.1480619.0590519.24750	20.88177
	18.82069	21.17282	21.5357219.7181719.9292421.3959219.0371619.0718719.12418	21.01025
	18.82395	21.13426	21.5996419.7280519.8203421.4717419.1985019.2520719.15598	20.87510
	17.23364	18.52025	19.1975617.4684817.9326319.1395016.1840417.5182117.22280	19.07494
	16.99212	18.92783	19.4563817.4357317.7526719.2411416.2405817.7841017.20077	19.03038
	16.78072	18.71101	19.2820917.5210017.7505019.2873415.7897117.9611517.20754	19.09152
	17.36035	18.30126	19.1263117.1479518.3175719.0351317.1251817.2423817.32717	18.87603
	17.13507	18.41935	19.3034217.2366818.0379519.0989417.1361217.1699417.43369	18.87275
	16.97080	18.33865	19.4392017.3436418.0302619.1049217.3199217.3656717.23421	18.88453
Temperature treatment	20.64974	24.20614	23.0326720.2313021.0332721.7946223.2499621.3300320.43881	21.72315
	20.45468	23.85209	22.6409120.9496420.7060121.8868023.3318221.3575020.40307	21.59085
	19.76102	23.13859	23.0553320.5554620.5282021.8153923.5337621.6183420.28239	21.70094
	19.27284	21.19419	21.8517719.6930719.8639020.6013120.5110819.3808019.21482	20.17071
	19.30109	21.36860	21.9876619.8351820.2098820.8628520.5074119.4106019.48065	20.16349
	19.13790	21.28010	22.0050519.6635920.0529720.7360320.4689219.4617619.30791	20.10378
	20.85570	23.08709	22.3571220.2000920.5159421.3850823.2216621.4670720.23438	21.52428
	20.45405	23.40426	22.1865920.2541020.3916521.3904823.1941121.3557720.18523	21.53250
	20.30248	23.15617	22.2406020.4565720.1740221.6157823.5383921.4888820.24817	21.52076

* Candidate reference gene.
